# DNA Methyltransferase HsdM Induce Drug Resistance on *Mycobacterium tuberculosis* via Multiple Effects

**DOI:** 10.3390/antibiotics10121544

**Published:** 2021-12-16

**Authors:** Hongqian Chu, Yongfei Hu, Bing Zhang, Zhaogang Sun, Baoli Zhu

**Affiliations:** 1 Translational Medicine Center Beijing Chest Hospital, Capital Medical University, Beijing 101149, China; chuhongqian0314@126.com; 2 Beijing Key Laboratory on Drug-Resistant Tuberculosis Research, Beijing Tuberculosis and Thoracic Tumor Institute, Beijing 101149, China; 3 CAS Key Laboratory of Pathogenic Microbiology & Immunology, Institute of Microbiology, Chinese Academy of Sciences, Beijing 100101, China; huyf@im.ac.cn; 4 Core Genomic Facility, Beijing Institute of Genomics, Chinese Academy of Sciences, Beijing 100101, China; zhangbing@big.ac.cn

**Keywords:** DNA methyltransferase, drug resistance, HsdM, *Mycobacterium tuberculosis*

## Abstract

Besides the genomic variants, epigenetic mechanisms such as DNA methylation also have an effect on drug resistance. This study aimed to investigate the methylomes of totally/extensively drug-resistant *M. tuberculosis* clinical isolates using the PacBio single-molecule real-time technology. The results showed they were almost the same as the pan-susceptible ones. Genetics and bioinformatics analysis confirmed three DNA methyltransferases—MamA, MamB, and HsdM. Moreover, anti-tuberculosis drug treatment did not change the methylomes. In addition, the knockout of the DNA methyltransferase *hsdM* gene in the extensively drug-resistant clinical isolate 11826 revealed that the motifs of GTAYN4ATC modified by HsdM were completely demethylated. Furthermore, the results of the methylated DNA target analysis found that HsdM was mainly involved in redox-related pathways, especially the prodrug isoniazid active protein KatG. HsdM also targeted three drug-targeted genes, *eis*, *embB*, and *gyrA*, and three drug transporters (Rv0194, Rv1410, and Rv1877), which mildly affected the drug susceptibility. The overexpression of HsdM in *M. smegmatis* increased the basal mutation rate. Our results suggested that DNA methyltransferase HsdM affected the drug resistance of *M. tuberculosis* by modulating the gene expression of redox, drug targets and transporters, and gene mutation.

## 1. Introduction

Tuberculosis (TB) is caused by the pathogen *Mycobacterium tuberculosis* (*M. tuberculosis*) and remains one of the leading causes of death caused by infectious pathogens, resulting in 10 million cases annually and latently infecting up to a third of the world’s population [1]. It is estimated that, in 2019, close to half a million people worldwide developed TB that was resistant to rifampicin (RR-TB), with 78% of these cases being multidrug-resistant TB (MDR-TB) [1]. The major mechanisms underlying *M. tuberculosis* drug resistance are mutations in chromosomal genes. Genotypic resistance results mostly from single-nucleotide polymorphisms (SNPs), insertions and deletions, and to a certain extent, deletions in genes that encode drug targets or drug-metabolizing enzymes within the bacilli [2,3]. The results of gene mutations include compensatory evolution, epistasis, clonal interference, decreased cell wall permeability, overexpression of efflux pumps, drug/target modification, and target mimicry, which enhances *M. tuberculosis* by modulating their fitness, enhancing their transmissibility, and stabilizing the resistance phenotype within their population [4,5].

Besides genomic variants, epigenetic mechanisms such as DNA methylation affect gene expression. Recently, studies based on whole-genome sequencing (WGS) of bacterial pathogens have provided novel insights into the evolution of *M. tuberculosis* drug resistance [6]. Despite many WGS studies, few reports exist about the epigenetic mechanisms of *M. tuberculosis* drug resistance. Bacterial DNA methyltransferases (MTases) can function as a restriction-modification system and are involved in various cellular processes, including the regulation of gene expression and antiviral defense [7,8]. Several pieces of evidence have revealed N6-methyladenine (m6A) and 5-methylcytosine (m5C) methylation mechanisms within *M. tuberculosis* genomes, and three DNA MTases, MamA, MamB, and HsdM, are responsible for m6A modification [9,10,11]. In particular, the MamA MTase, which targets the sequence motif CTCCAG, has been well characterized; the loss of MamA MTase can decrease gene expression and affect survival during hypoxia [9]. A recent study revealed 12 *M. tuberculosis* complex (MTBC) methylomes and analyzed the methylation at the genome level [10].

To date, no studies are available about the characterization of HsdM and its function in drug susceptibility and virulence. In this study, we sequenced the whole genome of seven *M. tuberculosis* strains, including four extensively drug-resistant (XDR) and two pan-susceptible clinical isolates and *M. tuberculosis* H37Rv (ATCC 27294), using high-precision PacBio single-molecule real-time (SMRT) sequencing technology [10] for their respective methylomes. In addition, the methylomes were tested with anti-tuberculosis drug treatment. Moreover, the response of MTases HsdM to drug resistance was investigated by HsdM gene knockout, combined with bioinformatics analysis and drug susceptibility tests.

## 2. Results

### 2.1. Genome Sequences of Clinical Strains of M. tuberculosis Using PacBio SMRT Technology

The WGS on clinical *M. tuberculosis* isolates (Table S1) was performed using the PacBio SMRT technology [12]. Six *M. tuberculosis* clinical isolates and a reference strain (H37Rv) were sequenced, and the sequencing coverage ranged from 80 to 240. The results of the general bioinformatics analysis are provided in Table 1. The genomic size ranged from 4,406,742 to 4,433,260 bp, and the gene number was predicted to range from 4420 to 4490. Compared with the sequencing reads obtained for the reference strain genome H37Rv (NC_000962) [13], 1650–1689 SNPs were found in clinical strains. Consistent with a previous study that showed that MTBC lineages displayed genomic diversity [14], we also found genetic variations among clinical strains and identified multiple SNPs (Table S3). All 44 SNPs, specific to modern Beijing isolates [15], were detected in the six sequenced clinical isolates (Table S3). Consistent with previous studies [10], HsdM in strain H37Rv contained an amino acid mutation, P306L, while intact HsdM and MamB were found in the present clinical isolates.

SMRT technology provides the possibility of direct identification of modified template nucleotides [12]. Taking advantage of SMRT technology, we found that 1948 adenines (99.08%) were modified at CTCCAG sites (modified by MamA). This was comparable with a previous study [10] that revealed 1947 modification sites in the H37Rv genome. In contrast, the CTGGAG/CTCCAG pair was not modified in the six sequenced clinical strains (Table 2). Consistent with another study [8,16], two other motifs—CACGCAG (predicted to be modified by MamB) and GTAYN4ATC (predicted to be modified by HsdM)—were also identified in the six sequenced clinical strains. The *M. tuberculosis* drug-resistant strain 10167 and the drug-susceptible strain 12058 had relatively low percentages of detected GATN4TTAC methylation motif (77.45% and 85.59%, respectively). The percentages of detected methylation for the CACGCAG motif were 99.15% and 95.91% in susceptible strains 12052 and 12058, respectively, which were almost the same as those of the drug-resistant strains. On the basis of DNA methylome analysis, MamA was found to be active in strain H37Rv, while HsdM and MamB were active in the six tested clinical isolates of *M. tuberculosis*.

### 2.2. Genome-Wide Kinetics of Adenine Methylation Did Not Correlate with Antibiotic Treatment

We examined methylated adenines in *M. tuberculosis* strain 11495 following treatment with individual antibiotics (INH, RIF, and LFX) using SMRT sequencing to test whether antibiotics affected the pattern of methylation at the genome level. In all cases, the methylated profiles remained largely unchanged following antibiotic stresses (Table S4). The genome-wide distribution of methylated bases was similar for treated and untreated cells (Table S4). These results implied that the MTase-mediated methylation of adenine was not inducible by antibiotics in *M. tuberculosis*.

### 2.3. Genome-Wide Identification of N6-Methyl-Adenine Base Modification by HsdM

**Figure 1:**
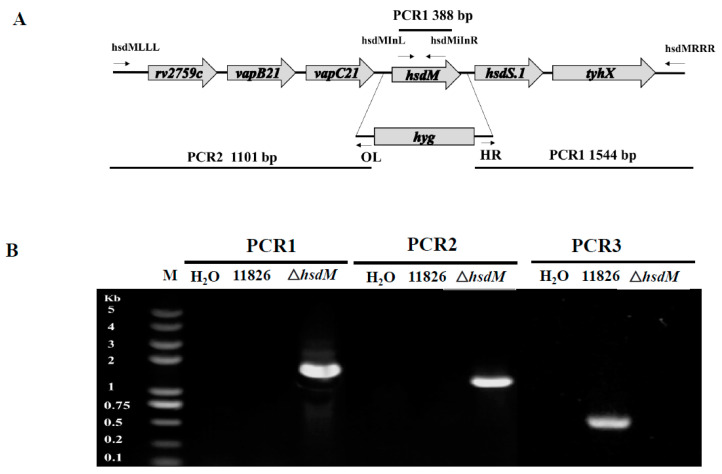
Generation of the 11826∆*hsdM M. tuberculosis* strain. (**A**) Genomic organization of the *hsdM* gene locus. Large arrows represent coding genes. Small arrows represent forward and reverse primers used for PCR. (**B**) PCR to confirm the loss of the *hsdM* gene.

We performed specialized transduction for deleting the *hsdM* gene to define the biological roles of HsdM in antibiotic susceptibility and pathogenesis. We failed to delete the *hsdM* in *M. tuberculosis* strain 11495, which might have been due to the lack of antibiotic selection or because *hsdM* was essential in strain 11495. However, we did successfully knockout the *hsdM* gene in the XDR strain 11826, and the mutant strain was designated 11826∆*hsdM*. The knockout of *hsdM* was confirmed by PCR (Figure 1). Unfortunately, we were unable to generate the *hsdM*-complemented strain from 11826∆*hsdM* due to the lack of an appropriate antibiotic marker.

**Figure 2:**
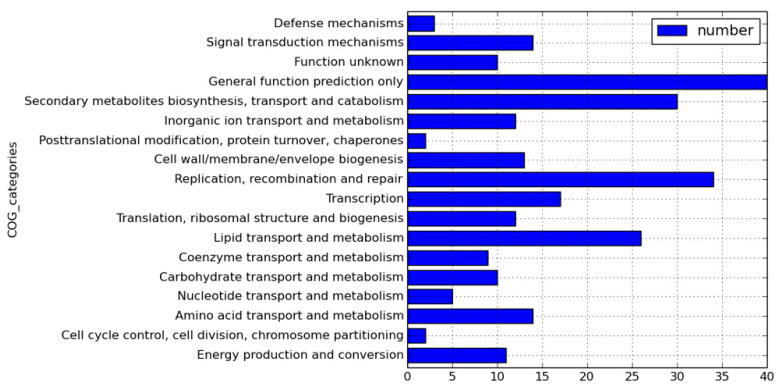
Functional classification of hsdM substrates.

**Figure 3:**
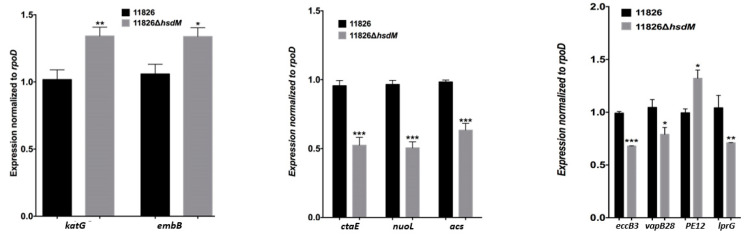
Genes are differentially expressed in the ∆*hsdM M. tuberculosis* strain. The expression of each gene was determined by quantitative real-time PCR in the ∆*hsdM* strain and its parental strain 11826, relative to the expression of an internal control gene, *rpoD*. Results are shown as the means ± standard derivations (SD) from three independent replicates. * *p* < 0.05; ** *p* < 0.01; *** *p* < 0.001.

We sequenced 11826∆*hsdM* using SMRT sequencing and compared methylated adenines between 11826∆*hsdM* and its parental strain 11826 to determine the HsdM substrates. The motif of GTAYN4ATC modified by HsdM was completely demethylated (Table S5). In 11826∆*hsdM*, the 355 motifs of GATN4ATC lost m6-methyl-adenine base modifications, including noncoding and coding sequences. We scanned the modified motif across the entire genome to assess the biological relevance of these proteins, and functional annotation clustering was used to cluster the HsdM substrate using our own scripts. Of the 335 motifs of GATN4ATC, 261 proteins were not limited to restriction-modification systems (Table S5), but covered 18 classification categories, such as replication, recombination and repair, transcription, translation, ribosomal structure and biogenesis, lipid transport and metabolism, and carbohydrate transport metabolism (Figure 2). The results suggested that HsdM affected a variety of biological functions. Further analysis showed that the substrates of HsdM were involved in the respiration pathway, the PE-PPE family, and drug resistance (Figure 2, Table 3 and Table S5), which were confirmed by the qPCR analysis of the mRNA levels of *CtaE*, *NuoI*, and *Acs* involved in the respiration pathway; *EccB3*, *VapB28*, *PE12*, and *LprG* belonging to the PE-PPE family; and *katG* and *EmbB* related to drug resistance (Figure 3).

### 2.4. HsdM Affected M. tuberculosis Growth *In Vitro*

**Figure 4:**
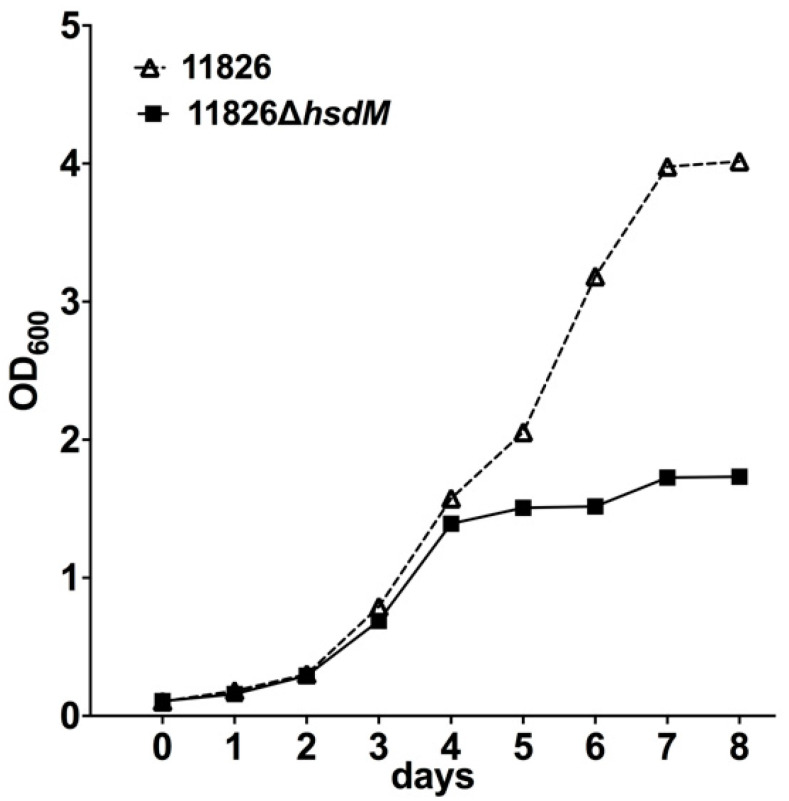
Growth rates of 11826∆*hsdM* and its parental strain 11826 in 7H9 medium. Data are presented as the means ± standard derivations (SD) from three independent replicates.

The effects of *hsdM* on growth were first investigated by comparing the growth rates of 11826∆*hsdM* and its parental strain 11826 in the 7H9 medium. The initial culture had an OD_600_ of 0.1, and the growth was monitored by measuring the OD_600_ over 7 days. No growth differences were detected within 4 days following inoculation (Figure 4). However, the growth of 11826∆*hsdM* was significantly slower (~2.3 lower) compared with the parental strain 11826 over longer incubation times. These results indicated that HsdM played a key role in *M. tuberculosis* growth in vitro.

### 2.5. HsdM Affected Drug Susceptibility

As bioinformatics analysis suggested that HsdM affected mycobacterial drug susceptibility, we then examined the drug susceptibility of *hsdM*-deficient *M. tuberculosis* in vitro. The MIC of the RIF, EMB, SM, and PAS for the 11826∆*hsdM* were 32 mg/L, 1 mg/L, 10 mg/L, 1024 mg/L, and 128 mg/L, respectively, the same as the corresponding values for its parental strain 11826. In addition, the MICs of the INH for 11826∆*hsdM* increased significantly, reaching four times those that of its parental strain 11826.

**Figure 5:**
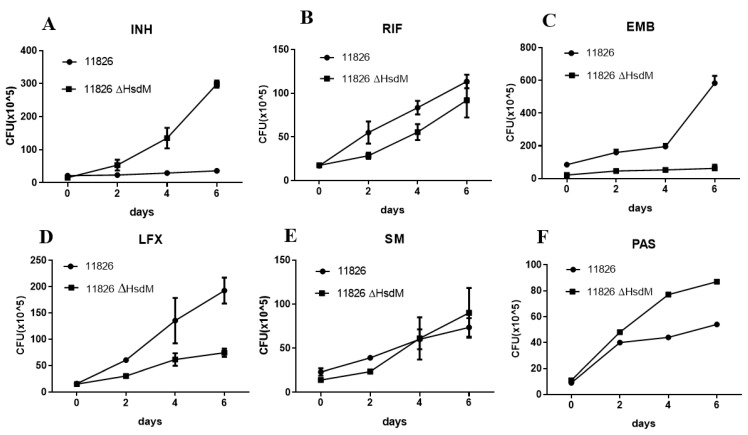
Changes in drug susceptibility in *M. tuberculosis* strains in the absence of the *hsdM* gene. Wild-type 11826 or 11826∆*hsdM* were grown in 7H9 medium to an OD_600_ of 0.1 and treated with INH at 6.4 mg/L (**A**), RIF at 16 mg/L (**B**), EMB at 10 mg/L (**C**), LFX at 0.5 mg/L (**D**), SM5 at 12 mg/L (**E**), and PAS at 664 mg/L (**F**). Survival was examined by monitoring colony-forming units at the indicated time points. Data are presented as the means ± standard derivations (SD) from three independent replicates. The figure presents the results of three biological replicates.

The *hsdM* knockout strain 11826∆*hsdM* and its parental strain 11826 were exposed to certain anti-tuberculosis drugs at the concentrations of half MICs of 11826∆*hsdM,* and the CFUs were measured at the time points of the early-phase growth to further determine the effect of HsdM on drug susceptibility. Following 6.4 mg/L INH treatment, 11826∆*hsdM* continued to grow, whereas no growth was detected for strain 11826. After 6 days of INH treatment, an approximate 1 log increase in cell growth was observed for 11826∆*hsdM* compared with its parental strain 11826 (Figure 5A). Similar to INH, a growth advantage of PAS treatment was found for 11826∆*hsdM* compared with its parental strain 11826 (Figure 5F). However, the strain 11826∆*hsdM* showed decreased growth compared with its parental stain 11826, following the treatment of Rifampin (RIF), levofloxacin (LFX), and Ethambutol (EMB) (Figure 5B–D), which was in accordance with their normal culture without the aforementioned anti-tuberculosis drugs.

### 2.6. Overexpression of HsdM Altered Mutation Rates in M. smegmatis

**Figure 6:**
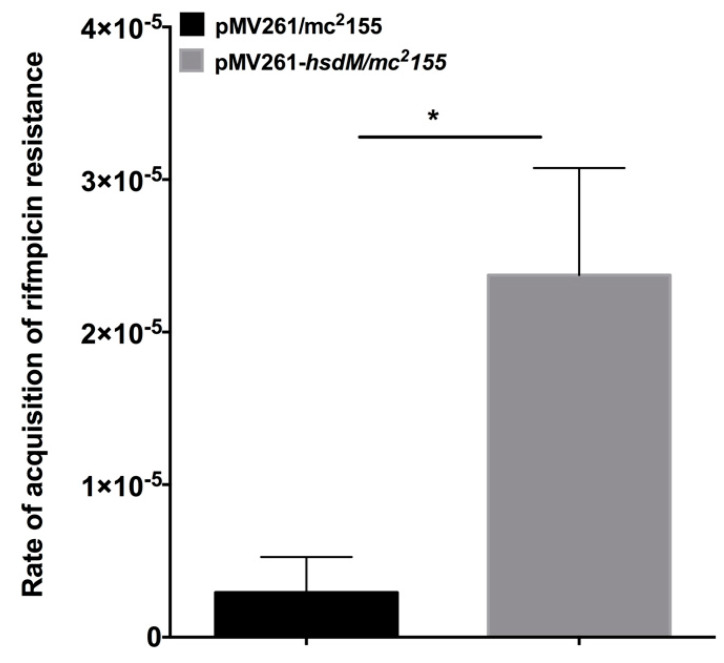
HsdM affected the rate of rifampicin resistance. The mutation rates of pMV261/mc^2^155 and pMV261-*hsdM*/mc^2^155 were indicated by the resistance to rifampicin. Black bar indicates pMV261/mc^2^155, and gray bar indicates pMV261-*hsdM*/mc^2^155. * *p* < 0.05.

*M. smegmatis* does not possess a homolog of HsdM. Therefore, we constructed the *hsdM* expression strain in this organism. We performed fluctuation analysis [17] on mycobacteria harboring pMV261-*hsdM* to explore whether HsdM affected the mycobacteria mutation rates. The mutation rate of the acquisition of rifampicin resistance in *M. smegmatis* harboring pMV261-*hsdM* was 2.3 × 10^−5^ ± 1.99 × 10^−5^ (*n* = 10) compared with 2.9 × 10^−6^ ± 2.3 × 10^−6^ (*n* = 10) in *M. smegmatis* harboring the pMV261vector alone (Figure 6). This result indicated that the overexpression of *hsdM* increased the basal mutation rate.

## 3. Discussion

In this study, we sequenced the methylomes of *M. tuberculosis* to single-base resolution using SMRT sequencing technology. Although the TDR/XDR *M. tuberculosis* clinical isolates showed no difference in methylome compared with the susceptible ones, and the tested anti-tuberculosis drugs had no obvious effect on genome DNA methylation, the knockout of HsdM in the XDR clinical isolates of strain 11826 indicated its modulatory roles in bacterial antibiotic susceptibility, growth curves, and bacteria redox status, which had a profound effect on the drug resistance of *M. tuberculosis*.

Two TDRs, two XDRs, and two pan-susceptible strains were sequenced to investigate whether the drug resistance patterns of the clinical isolates affected the methylome; almost the same number and pattern of the methylated motifs were found in these six isolates. Further antibiotic treatment with OFX, RIF, and INH did not affect the N6-methyl-adenine base modification under drug treatment (Table S3), which was consistent with the stable methylation levels in antibiotic stress in *E. coli* [18]. The frequency of methylated sequences among these motifs was nearly 100% in almost all the tested drug-resistant or drug-sensitive clinical isolates in this study (Table 2), suggesting that the MTases recognizing these three DNA patterns were all active [19]. However, in some drug-resistant clinical isolates, only a fraction of the motifs recognized by MTases were methylated, implying that the activity of these MTases was reduced, but not eliminated [20]. Different numbers and fractions of methylated motifs might affect the bacterial growth in the medium. The number of hsdM-methylated motifs is almost double in BCG (674) than in clinical XDR-TB isolates 11826 (368). In comparison to their parent strain, the knockout of hsdM in BCG did not reveal a growth difference, while the knockout of hsdM in XDR-TB 11826 showed a drop in growth [21]. There were also some reports on the HsdM homolog in other bacterial species, such as in streptococci (e.g., S. pyogenes and S. agalactiae), which might involve bacterial drug resistance and other physiological features such as growth, hypoxia, and UV stress [22,23].

Differential methylated genes were negatively correlated with their transcriptional levels in rifampicin- or isoniazid-resistant strains [20], such as Rv0840c, Rv2243, Rv0644c, Rv2386c, and Rv1130 in rifampicin-resistant strains and Rv0405, Rv0252, and Rv0908 in isoniazid-resistant strains. Moreover, the methylation of the promoters of sigma factors exhibited indirect mechanisms of expression regulation [19]. Several drug target genes (*gyrA*, *embB*, and *eis)* and drug transporters (*Rv0194*, *Rv1410c*, and *Rv1877*) were found to be methylated by MTase HsdM (Table 3 and Table S5), and statistically significantly different expression was detected in 11826∆*hsdM* compared with its parental strain 11826. We confirmed that the mRNA level of *katG* and *embB* statistically significantly increased in the 11826∆*hsdM* mutant strain compared with the parental strain 11826 (Figure 3). These results indicated that DNA methylation regulated the drug resistance at transcriptional levels.

The INH-activated catalase-peroxidase KatG [24] was not found in the N6-methyl-adenine base modification. However, it was reported that the widespread promoter methylation, including the INH-resistance-related genes, did affect the differential expression in the ∆hsdM transcriptome [25]. Previous studies suggested that the ratio of bacterial susceptibility to INH was affected by nicotinamide adenine dinucleotide (NAD/NADH) [26,27]. Indeed, several HsdM substrate gene-encoding proteins were classified within the category of cellular respiration. These encoding proteins might interfere with the NAD/NADH ratio, thus further affecting INH susceptibility. An investigation of the antibiotic susceptibility profile of 11826∆*hsdM* showed that 11826∆*hsdM* was more resistant to INH than its parental strain. Further analysis of the biological function of HsdM revealed that the methylation might affect the drug resistance via the expression of some substrates involved in the redox-related pathway (Figure 2 and Table S5), which was in accordance with MamA involved in the stress condition of hypoxia [9]. These results suggested that HsdM affected the drug susceptibility indirectly by interfering with the mycobacteria redox status rather than with the gene expression regulation.

Similar to the study that revealed the decreased survival of *E. coli* following the knockout of the Dam MTase-encoding gene after bactericidal ß-lactam antibiotic treatment, our results showed that the survival of 11826∆*hsdM* was compromised when individually treated with bactericidal antibiotics RIF and LFX (Figure 5). In contrast, following individual treatment with the bacteriostatic antibiotics INH and PAS, the survival of 11826∆*hsdM* increased compared with its parental strain 11826 (Figure 5). Therefore, bacteriostatic and bactericidal antibiotic treatments led to different outcomes, inhibiting bacterial growth or killing bacteria. These two types of drugs both interfered with the redox status of cells by targeting the respiratory pathway [28,29]. We could not measure to what extent the delayed growth of the 11826∆*hsdM* strain (Figure 4) affected the bacterial survival after drug treatment compared with that of its parental strain 11826. However, we confirmed that MTase HsdM could partially contribute to the higher drug-resistant mutant rate in the HsdM-overexpressing strain (Figure 6).

In this study, only a limited number of clinical isolates were tested, and no comparison was conducted in terms of genome methylation among the drug-sensitive, multidrug-resistant, and XDR clinical isolates. Additionally, the hsdM and its related regulation genes such as hsdS and hsdS.1 were neglected. Moreover, we did not establish a Wayne model of hypoxia to check the expression levels of latent genes in 11826∆hsdM and its parent strain so as to know whether the XDR strain has a different expression level compared to the BCG strain, or whether they are the same, under hypoxic conditions.

## 4. Conclusions

In summary, the DNA methylome profiles in drug-resistant clinical isolates were almost the same as those in pan-susceptible ones. The functional effects of MTase HsdM in *M. tuberculosis* clinical isolates could affect the drug resistance via different methylation pathways, such as the drug target genes, genes involving the redox-related pathway, and drug resistance mutations.

## 5. Materials and Methods

### 5.1. Bacterial Strains and Culture Conditions

All information relating to the mycobacterial strains used in this study is listed in Table S1. In particular, the *M. tuberculosis* strains 11495 and 10167 were found to be totally drug-resistant (TDR), displaying resistance to all tested antibiotics, including isoniazid (INH), rifampicin (RIF), ethambutol (EMB), streptomycin (SM), amikacin (AmK), levofloxacin (LFX), para-aminosalicylic acid (PAS), and capreomycin (CPM), tested by the agar dilution method in the clinic. The *M. tuberculosis* strains 11776 and 11826 are XDR strains. However, the *M. tuberculosis* strains 12052 and 12058 are drug-susceptible strains. All these clinical mycobacterial isolates and *M. tuberculosis* H37Rv strains (ATCC27294) were stored at the Beijing Chest Hospital and grown in Middlebrook 7H9 with 10% OADC (oleic acid, albumin, dextrose, and catalase; BD, Sparks, MD, USA), 0.5% (*v*/*v*) glycerol, and 0.05% (*v*/*v*) Tween 80 for further use. The growth curves of the *M. tuberculosis hsdM* mutant strain 11826∆hsdM and its parental strain 11826 were compared in 7H9 medium by monitoring the OD_600_ at different time points.

### 5.2. Generation of the hsdM-Knockout Mutant Strain

Mycobacteriophage-based specialized transduction was explored for the replacement of the *hsdM* gene as previously described [30]. The upstream and downstream sequences were amplified from the genomic DNA of the *M. tuberculosis* clinical strain 11826. The corresponding primers for the 11826∆hsdM mutant strain are listed in Table S2, and the corresponding positions are indicated in Figure 1. Plasmid p004-*hsdM* was derived from pYUB1471 and contained the upstream and downstream regions of the *hdsM* gene. The linearized plasmid p004-*hsdM* with *Pac*I was inserted into the phage vector phAE159 for further phage packaging using the MaxPlax packaging extract (Epicentre, Madison, WI, USA) and transformed into Escherichia coli HB101 cells. After propagation in *M. smegmatis* mc^2^155, the phage was transfected into individual *M. tuberculosis* strain 11826 cells, and the correct transformants were confirmed by polymerase chain reaction (PCR) using the primer pair hsdMInL/hsdMInR. This study focused on drug resistance; therefore, we tested the TDR/XDR clinical isolates. Unfortunately, we could not generate the *hsdM*-complement strain from 11826∆*hsdM* due to the lack of an appropriate antibiotic marker.

### 5.3. Antibiotic Susceptibility Testing

The minimum inhibitory concentrations (MICs) of 11826∆*hsdM* and its parental strains to certain antibiotic drugs (INH, RIF, EMB, SM, PAS, and LFX; Sigma–Aldrich, St. Louis, MO, USA) were determined on microplates using the alamarBlue modified microplate assay as previously described [31]. Briefly, approximately 10^5^ cells/well were incubated for 7 days at 37 °C in the 7H9 medium plus OADC with different diluted concentrations of drugs. The color changes from blue to pink were recorded after the indicator (0.02% resazurin) was added to individual samples for 48 h.

The bacterial cultures (OD_600_ ~ 0.1) were treated with drugs at the indicated concentrations of half MICs of 11826∆*hsdM* to further find the drugs for the early-phase growth. The number of colony-forming units (CFUs) was measured at the indicated time point. Experiments were performed in triplicate.

### 5.4. SMRT Sequencing

Genomic DNA from mycobacterial strains was extracted using a Qiagen kit (Genome DNA buffer set 19060, Hilden, Germany) and sequenced using the Pacific Biosciences RSII DNA sequencing system (Pacific Biosciences, Menlo Park, CA, USA), with a 10-kb SMRTbell library, following the manufacturer’s recommendations, and with an additional bead clean-up step before primer annealing. The library was bound with P4 polymerase, and the subsequent complexes were loaded onto version V3 SMRT cells. Each sample was sequenced on one or two SMRT cells to give a genome coverage of >100-fold per sample.

### 5.5. Bioinformatics Analyses

Genome assembly was explored using the Hierarchical Genome Assembly Process (HGAP.3) algorithm in the SMRT Portal (version 9.0.0, Pacific Biosciences, Menlo Park, CA, USA) and further corrected with the Quiver algorithm software [32]. Standard settings (QV > 30) in the “RS_Modification_and_Motif_Analysis.1” protocol included in the SMRT Portal version 2.2.0 were used for detecting base modifications and the sequence motifs. Then, the genome annotation was conducted via Rapid Annotation using Subsystem Technology (RAST) [33]. Finally, the functions of predicted protein-coding genes were annotated by means of comparisons with the NCBI-NR and Clusters of Orthologous Genes (COG) databases.

### 5.6. De Novo Assembly Details

The sequence data of type I, type II, and type III M genes were downloaded from Rebase (http://rebase.neb.com/rebase/rebase.seqs.html (accessed on 2 November 2021)) to search for specific DNA methylation genes. Then, we aligned the assembled sequences against the Rebase data using the Basic Local Alignment Search Tool (BLAST) (identity > 90%; coverage > 90%) to identify MTase genes.

### 5.7. RNA Isolation and Quantitative Real-Time PCR

After collection by centrifugation at 12,000× *g*, the bacteria pellets were suspended in TRIzol (Invitrogen, Carlsbad, CA, USA). RNA was then purified following the manufacturer’s protocols. cDNA was synthesized using the SuperScript III first-strand synthesis system (Invitrogen). The *M. tuberculosis* DNA-directed RNA polymerase α subunit *rpoD* gene was used as a control to normalize gene expression tested by qRT-PCR in a Bio-Rad iCycler (Bio-Rad, Hercules, CA, USA). The 2^−∆∆CT^ method [34] was used to calculate the relative gene expression in mycobacteria. The primers used are described in Table S2.

### 5.8. Mutant Calculation

When the OD_600_ reached 0.5, the mycobacterial cultures were diluted using 7H9 supplemented with 10% ADS (5% (*w*/*v*) (bovine serum albumin fraction V, 2% (*w*/*v*) dextrose, and 8.1% (*w*/*v*) NaCl)), 0.5% (*v*/*v*) glycerol, and 0.05% (*v*/*v*) Tween 80, to a final concentration of 10,000 cells/mL. Then, 0.5 mL with about 1000 bacilli was inoculated into 10 mL of the culture medium. The CFUs of the initial inoculation were determined by serial dilution and plating on LB medium. After 48 h of incubation, the CFUs of the corresponding cultures were also determined by serial dilution and plating on LB medium with RIF. The mutation rate was calculated using the following formula: *a* = [2ln 2(*Mt*/*Nt*–*M*_0_/*N*_0_)]/*n*. *M*_0_ indicates mutants at time 0, *Mt* indicates the number of mutants at time *t*, *n* indicates the number of generations, *N*0 indicates the cell number at time 0, and *Nt* indicates the cell number at time *t*.

## Figures and Tables

**Table 1:** General genome information of sequenced *M. tuberculosis* strains.

Strain Names	Average Read Size (kb)	Sequencing Depth (x)	Genome Size (bp)	Gene Number	SNP	Indel
***M. tuberculosis* 11495**	3.9	180	4,428,395	4455	1687	392
***M. tuberculosis* 10167**	2.1	240	4,418,815	4481	1677	501
***M. tuberculosis* 11776**	3.5	120	4,433,260	4501	1685	488
***M. tuberculosis* 11826**	2.5	160	4,406,742	4527	1689	619
***M. tuberculosis* 12052**	2.4	150	4,421,905	4538	1655	522
***M. tuberculosis* 12058**	2.7	90	4,425,864	4490	1650	459

**Table 2:** Profile of the N6-methyl-adenine base modifications in sequenced *M. tuberculosis* strains.

Strain Name	Methylated Motif					
	CTCCAG/CTGGAG		CACGCAG		GATN4TTAC	
	No. of Motifs Detected	% Motifs Detected	No. of Motif Detected	% Motifs Detected	No. of Motifs Detected	% Motifs Detected
H37Rv	99.08	1930	/	/	/	/
11495	/	/	99.41	839	97.84	363
10167	/	/	100	828	77.45	285
11776	/	/	99.16	825	98.4	362
11826	/	/	99.27	826	96.45	355
12052	/	/	99.15	825	94.29	347
12058	/	/	95.91	798	85.59	315

**Table 3:** Selected gene loss of methylated modification in *M. tuberculosis* 11826∆*hsdM.*

Gene Name	Function
Respiration-related genes	
*ctaE*	Involved in aerobic respiration, probable cytochrome c oxidase (subunit III) CtaE
*qcrC*	Probable ubiquinol-cytochrome c reductase QcrC (cytochrome c subunit)
*NuoI*	Involved in aerobic/anaerobic respiration
*cyp126*	Cytochrome P450 126 Cyp126, involved in intermediary metabolism and respiration
*cyp135B1*	Cytochrome P450 135B1 belongs to a group of heme-thiolate monooxygenases
*fgd1*	Catalyzes the oxidation of glucose-6-phosphate to 6-phosphogluconolactone using coenzyme F420 (an *-hydroxy-5-deazaflavin derivative) as the electron acceptor
*frdB*	Involved in the interconversion of fumarate and succinate (anaerobic respiration)
*fdhF*	Decomposes formic acid to hydrogen and carbon dioxide under anaerobic conditions in the absence of exogenous electron acceptors
*Rv1786*	Ferredoxin, an iron-sulfur protein that transfers electrons in a wide variety of metabolic reactions; involved in intermediary metabolism and respiration
*qor*	Rv1454c, a quinone reductase
Lipid metabolism-related genes	
*fadD11*	Rv1550, fatty-acid-CoA ligase
*fadD12*	Rv1427c, long-chain-fatty-acid—CoA ligase, function unknown, but supposed involvement in lipid degradation
*fadD16*	Rv0852, possible fatty-acid-CoA ligase FadD16, function unknown, but involved in lipid degradation
*fadD2*	Rv0270, probable fatty acid-CoA ligase, function unknown, but involved in lipid degradation
*fadD23*	Rv3826, long-chain-fatty-acid—CoA ligase
*fadD24*	Rv1529, long-chain-fatty-acid—CoA ligase
*fadD29*	Rv2950c, long-chain-fatty-acid—CoA ligase
*fadD35*	Rv2505c, long-chain-fatty-acid—CoA ligase
*fadE10*	Rv0873, probable acyl-CoA dehydrogenase, function unknown, but involved in lipid degradation
*lipL*	Rv1497, probable esterase, function unknown, but supposed involvement in lipid metabolism
Drug resistance-related genes	
*gyrA*	Rv0006, DNA gyrase subunit A, related to fluoroquinolone resistance
*eis*	Rv2416c, enhanced intracellular survival protein, related to kanamycin resistance
*embB*	Rv3795, arabinosyltransferase B, related to ethambutol resistance
*Rv0194*	Transmembrane multidrug efflux pump, related to multidrug resistance
*Rv1410c*	EmrB/QacA family drug resistance transporter, related to aminoglycosides/tetracycline resistance
*Rv1877*	EmrB/QacA family drug resistance transporter

## Data Availability

The data presented in this study are available upon request from the corresponding author.

## Supplementary Materials

The following are available online at https://www.mdpi.com/article/10.3390/antibiotics10121544/s1, Table S1: The susceptibility of tested drugs in bacterial strains used in this study. Table S2: Primers used in this study. Table S3: SNPs identified in the sequenced M. tuberculosis strains. Table S4: Profile of the N6-methyl-adenine base modifications in M. tuberculosis strains exposed to drug treatment. Table S5: HsdM substrates identified in M. tuberculosis strain 11826.
